# Editorial: Late-life psychopathology

**DOI:** 10.3389/fpsyg.2023.1204202

**Published:** 2023-05-25

**Authors:** Mithat Durak, Selin Karakose, W. Quin Yow

**Affiliations:** ^1^Department of Psychology, Bolu Abant Izzet Baysal University, Bolu, Türkiye; ^2^College of Medicine, Florida State University, Tallahassee, FL, United States; ^3^Humanities, Arts and Social Sciences, Singapore University of Technology and Design, Singapore, Singapore

**Keywords:** older adults, late-life depression, late-life anxiety, social isolation, dementia, psychological trauma, schizophrenia, sleep disorders

The Research Topic, “*Late-life psychopathology*,” concerns how psychopathology might present itself in old age. The collection includes a broad spectrum of older individuals' behavioral, cognitive, and emotional issues, including mood disorders, anxiety disorders, psychotic disorders, sexual disorders, insomnia, and personality disorders. Along with the psychopathological disorders in older adults, this Research Topic focused on the psychopathological similarities and differences across the various age groups.

Psychological health issues are particularly challenging for the older. Alzheimer's disease and dementia, depression, anxiety, and intense loneliness are just a few of the psychological health problems burdened with negative consequences in old age. However, many older persons encounter difficulties seeking proper psychological resources and support. Healthcare professionals need to comprehend the different mental health issues that older individuals confront to provide them with the finest available treatment.

Depression is one of the most prevalent mental health issues among older adults, and it is associated with poor physical health and a lower quality of life. Anxiety is another prevalent issue among older adults and can be attributed to changes in lifestyle, such as retirement or deteriorating physical health. Age-related or other biological factors, such as Alzheimer's disease, can cause cognitive impairment, including memory problems, problem-solving difficulty, and other thought processes challenges. These psychological disorders may have a substantial impact on the persons afflicted as well as their close family members.

It is essential to comprehend how these psychological disorders manifest to develop treatment strategies that meet the needs of older individuals. In addition to delineating psychopathological issues in older adults, the articles in this Research Topic collection offer future recommendations for mental health professionals and scientists.

Psychological problems in older adults are often overlooked and untreatable because clinicians believe psychological disorders are either a sign of a physical ailment or a natural consequence of aging (Durak, 2018). While the Diagnostic and Statistical Manual of Mental Disorders 5^th^ Edition (DSM 5; American Psychiatric Association, 2013) acknowledges that a major depressive episode may be the first sign of irreversible dementia in many older adults, it also emphasizes that memory problems often resolve when the major depressive episode is treated effectively. Thus, it is crucial to comprehend how various health issues present and interact with one another to create effective treatment strategies that cater to the requirements of older people.

Besides, chronic physical diseases and psychological disorders interact negatively and exacerbate each other's severity, making treatment more difficult in older adults. Furthermore, exposure to high-stress levels renders older adults more vulnerable to psychopathology than the different age groups. For example, older adults have been disproportionately affected by the COVID-19 Pandemic (Durak and Senol-Durak, 2020), resulting in many individual and negative social consequences around the world as a result of the days-long quarantine, social isolation, limited ability to leave the house, vulnerability to the virus due to physiological health problems, and high virus-related mortality ratio.

Psychopathology can appear differently in older adults than in younger adults. Specialists attempting to conduct a psychological assessment or diagnose such disorders in older adults should be more cautious and thorough than in other age groups. These include changes in sleep patterns or difficulty falling or staying asleep; withdrawal from one enjoyable activity; significant apathy or lack of energy; confusion, disorientation, and difficulty making decisions; difficulty expressing themselves, difficulty following a conversation, or impaired speech; anxiety, paranoia, or delusional thinking; irritability or mood swings; and memory loss or confusion between recent and distant events. According to the DSM-5, in contrast to other age groups, older adults' agoraphobia is associated with fear of falling and incontinence, and little is known about “body dysmorphic disorder” despite its prevalence among older adults. In this context, the Research Topic “*Late-life psychopathology*” encourages cross-sectional and longitudinal comparative studies on the types, assessment, and treatment of psychopathology in older adults, as well as on the contributory variables associated with the presence of psychopathology in older adults.

Psychopathology at a later age is often characterized by a complex interaction of several co-occurring disorders, such as dementia, social isolation and loneliness, anxiety and mood disorders, depression, psychological traumas, schizophrenia, and even substance abuse (Bernacchio et al., 2009). For instance, insomnia symptoms are associated with depressive symptoms and suicide risk in older adults; suicidal older adults tend to misuse substances (Webb et al., 2018); and physical dependency with bereavement increases the risk of suicide (De Leo, 2022). Justice-involved older adults are more likely to have or develop mental illness and substance use disorders (Han et al., 2021). Recent discussions have focused on the importance of previous events in one's life. For instance, older adults with substance use disorders are more likely to be abused than those without the disorders (Mercier et al., 2020). Besides, sexual assault and emotional abuse may play a role in the development or experience of aging-related genitourinary dysfunction in older women (Gibson et al., 2019).

This collection of articles focuses on the types, assessment, and treatment of psychopathology in older adults, contributory variables associated with the presence of psychopathology in older adults, and psychopathological similarities and differences across the various age groups. For example, social isolation and loneliness in older adults tend to be common descriptions of older adults' quality of life and generally negatively affect older adults' wellbeing and health (Gardiner et al., 2018; Smith and Lim, 2020; Smith et al., 2020). Social isolation is especially so in light of our global pandemic (Berg-Weger and Morley, 2020; Smith et al., 2020; Wong et al., 2020; Kasar and Karaman, 2021; Kotwal et al., 2021; van Tilburg et al., 2021).

It is reported that 50% of adults over sixty are at risk of social isolation, and nearly one-third of the older adult population experiences loneliness and social isolation (Berg-Weger and Morley, 2020; Fakoya et al., 2020). Feelings of loneliness also tend to be linked to depression and other mental health problems (Calati et al., 2019; Lee et al., 2019). It is frequently emphasized by several researchers that older adults are more likely to feel lonely and socially isolated (Fakoya et al., 2020; Smith et al., 2020; Rentscher et al., 2021). Those feelings have arisen during the pandemic outbreak (Rodrigues et al., 2022), particularly during the acute phase of the epidemic (Luchetti et al., 2021). Recently, several researchers have discussed the applicability of technology to reduce social isolation (Sen et al., 2022), while others have highlighted the value of face-to-face communication (Su et al., 2023).

Still, many cultural and social factors could help older adults deal with these problems and improve their quality of life. In one of the articles in this issue, the authors looked at whether or not intergenerational relationships could help older people feel less alone and have a more positive view of getting older by testing the mediational role of intergenerational relationships between a sense of loneliness and a positive attitude toward later life (Liu et al.). They found that the overall quality of intergenerational relationships was positively related to older parents' attitudes toward later life but partially mediated by a sense of loneliness. A systematic review and meta-analysis of remotely delivered interventions on loneliness in older adults by the authors of another paper suggest that such timely interventions could help reduce loneliness in older adults through a systematic review and meta-analysis of remotely delivered interventions. Nonetheless, it may be altered by media type, treatment strategy, participant characteristics, measurement time points, and other variables.

Under the “*Late-life psychopathology*” topic, researchers were invited to submit manuscripts about the diverse types of psychopathological disorders and the psychological assessment of psychopathology in older adults. The Research Topics are articles about the onset, development, prevalence, and assessment of psychopathological disorders and related problems in old age. The following are some of the main themes recommended but not limited to mood disorders, anxiety disorders, death anxiety, psychotic disorders, suicide, complex traumas, mourning, sexual dysfunctions, insomnia, substance use, suicide, social isolation-loneliness, and neglect-abuse-violence against older adults.

The Research Topic, late-life psychopathology, has thirteen published articles. Ten of the thirteen papers are “original research,” with the other three falling into one of three categories: “perspective,” “opinion,” or “systematic review.”

The first article by Xiang Y. et al., entitled “*Delays in Seeking Medical Services in* ***Elderly*** *Patients with Senile Cataract,”* is an original research article that determines how often people with senile cataracts delay visiting the doctor and receiving treatment, as well as uncovers any relevant consequences or risk factors. According to the authors, the findings of this research may assist physicians in better understanding and treating psychopathology in older people. Longevity benefits humanity but introduces new challenges to healthcare and society. However, postponing medical appointments and treatment is a widespread and critical issue among seniors, resulting in disease progression and a worse prognosis. This research evaluated the prevalence of delaying medical visits and treatment, visual impairment, life inconvenience, perceptions of disease treatment, and associated factors in individuals with senile cataracts. A total of four hundred patients, ages 60 to 94, are enrolled. A considerable proportion of older individuals with senile cataracts delay medical visits (73.5%) and surgical treatment (74.5%), impairing their ability to lead a normal life. Almost half have erroneous beliefs about cataract therapy. The authors recommend that the public pay more attention to the health of older and younger individuals, as well as their senior relatives.

The second article by Fu et al., entitled “*The Effectiveness of Remote Delivered Intervention for Loneliness Reduction in Older Adults: A Systematic Review and Meta-Analysis,”* is an original research article that conducts an updated meta-analysis and systematic review to assess the efficacy of a remotely delivered intervention for loneliness in older people using randomized controlled trials (RCTs). A wide variety of mental health issues, including depression, schizophrenia, and psychotic disorders, are examined by remote-delivered intervention. Following a comprehensive search of major databases, thirteen articles are reviewed. The findings support the idea that a remotely delivered intervention may help older people feel less lonely; however, the effectiveness is impacted by factors such as media type, treatment strategy, and group format. This study indicates the value of remote-delivered intervention in reducing loneliness and warrants further investigation into its use. With little modifications, these interventions may be compatible with COVID-19 shielding/social distancing strategies and help older people overcome loneliness.

The third article by Liu et al., entitled “*Intergenerational Relationship Quality and Attitude toward Later Life among Aging Chinese Adults in Hong Kong: The Mediating Role of the Sense of Loneliness,”* is an original research article that assessed the quality of intergenerational relationships using a four-dimensional framework: Affectual intimacy, structural-associational solidarity, consensual-normative solidarity, and intergenerational conflict. They examined the influence of intergenerational connection quality on older adults' perspectives about later life as evaluated by feelings of isolation. The effects of structural-associational solidarity and intergenerational conflict on attitudes toward later life were almost totally mediated by a feeling of loneliness. In contrast, the effects of consensual-normative solidarity and affectual closeness were only somewhat mediated. The research reveals that isolation among older individuals is a modifiable risk factor that needs greater consideration in social policies and services and might be mitigated within the family context.

The fourth article by Xiang X. et al., entitled “*Childhood adversity and cognitive impairment in later life,”* is an original research article that intends to investigate the association between childhood adversity and cognitive impairment in older adults using longitudinal data from the Health and Retirement Study (HRS; 1998–2016 surveys), as well as the potential moderating influences of gender, race, and education in a population-based sample of older Americans. The authors noted that they were motivated to conduct the study because empirical evidence is equivocal on whether the detrimental impact of childhood adversity on cognition in early life survives into later adulthood, with contradictory results in the existing literature. The researchers concluded that there is variance across gender and ethnicity, with some aspects of childhood adversity continuing to impair cognitive function in later life (e.g., grade retention). In contrast, other events may have the reverse effect.

The fifth article by Patel, entitled “*Vulnerability as determinant of suicide among older people in the Northern Indian States,”* is an original research article that discusses the nature of suicide among older people. This study's data was gathered from news articles published in Indian newspapers, magazines, and online news portals between March and June 2022, focusing on sixty cases of senior citizens who committed suicide in the northern Indian states of Bihar, Delhi, Haryana, Madhya Pradesh, Maharashtra, Rajasthan, Uttar Pradesh, and Uttarakhand. According to the study, the number of older people who commit suicide increases significantly as they age and become more vulnerable. According to the study, older people are more likely to commit suicide because of personal, family, and societal problems. This vulnerability has been shown to negatively affect not just suicidal behavior but also family abuse, chronic disease, depression, poverty, and social rejection, all of which are associated with suicidal behavior.

The sixth article by Castro-de-Araujo et al., entitled “*Patterns of multimorbidity and some psychiatric disorders: A systematic review of the literature,”* is a systematic review article that investigates the association between five prevalent psychological disorders (such as depression, anxiety, PTSD, substance use disorder, and psychosis) and non-psychiatric diseases (such as tuberculosis and HIV), as well as the pattern of association between them. Because the influence of psychological problems on people with non-psychiatric chronic illnesses is not well known, this is a comprehensive review of research published in multimorbidity since 2015, including both psychological disorders and chronic diseases. After reviewing fifty-six articles, only twenty-six were selected for inclusion in the study, and it was determined that there are strong associations between depression, psychosis, and multimorbidity. This study presents an overview of the multiple morbidity paradigm, emphasizing mental illness and probable prospects, which is important for managing the processes associated with multiple morbidity patterns.

The seventh article by Aisenberg-Shafran, entitled “*Psychotherapy for late-life psychopathology–updates to promote aging in place,”* is a perspective article. The goal of the study discussed in this article is to look at the diverse ways to improve older people's psychological wellbeing. A key point of this article is that even if psychopathology in older adults does not fulfill the diagnostic criteria for mental disorders, it will nonetheless have repercussions for the affected person, as well as for their loved ones, the workplace, and the larger community. It is insufficient to rely on psychologists, social workers, or gerontologists to improve older people's mental health. This global commitment requires psychoeducation at all levels of society, from children and teens to older people, medical experts, government officials, and lawmakers. Furthermore, the study suggests that community-based psychotherapy clinics be established inside academic institutions to serve local populations better. As indicated by higher suicide rates among older adults, a sizable portion of the population could benefit from psychotherapy in their later years but does not get help. Difficulty in finding help in easily accessible hospitals is a daunting task due to the stigma surrounding those who deal with mental illness and the negative preconceptions associated with psychotherapy and other forms of psychological treatment based on outdated ideas. Some of the recommendations in the paper include offering low-cost, high-quality psychotherapy to older adults, with the goals of improving their wellbeing as they age and helping them adjust to major life changes (like retirement, widowhood, declining health, and loss), helping family members through the challenges of caregiving or aging alongside a loved one, and building professional and therapeutic training with an emphasis on working with the older adult population.

The eighth article by Zhou et al., entitled “*The effects of aging and perceived loneliness on lexical ambiguity resolution,”* is an original research article focusing on the social factors associated with the language process in older adults. Besides investigating the effects of aging with a performing control group, this study also examined the perception of loneliness on lexical ambiguity resolution. Findings revealed that older adults performed greater lexical effects than younger adults but had similar sub-lexical ambiguity. The results of this study also demonstrated that higher perceived loneliness was associated with displaying a greater sub-lexical ambiguity disadvantage effect. This study highlighted the important link between social connections and language processing in older adults.

The ninth article by Choi and Marti, entitled “*Intent disclosure in late-life suicide: age group differences in correlates and associations with suicide means,”* is an original research article that examines associated factors with the disclosure of intent to die by suicide across three age groups (65–74, 75–84, and 85+) of older suicide decedents using 2017–2019 data from the United States National Violent Death Reporting System (NVDRS). Aside from having important preventive implications, the study's attention has been relatively under-examined in prior publications. The authors note that the suicide incidence among older adults continues to climb, especially among males aged seventy-five and over, and that there is a dearth of research into effective interventions for preventing suicide in older adults. Additionally, they recommend interacting with older people who express suicidal ideation as a means of suicide prevention. The findings provide insights into the demographic and clinical characteristics of older-adult suicide decedents who disclosed their suicide intent and have important clinical implications for reducing premature mortality from suicides.

The tenth article by Hafford-Letchfield et al., entitled “*Talking really does matter: Lay perspectives from older people on talking about suicide in later life,”* is an original research article investigating potential barriers and enablers in discussing suicidal tendencies from the perspectives of lay older people. Fifteen in-depth interviews with participants aged 70–89 are performed and examined thematically. The data collection method (in-depth interviews) is advantageous since it allows research participants to express themselves freely and yields abundant data to better comprehend the phenomena under investigation. The findings illustrate the potential for involving older people themselves and people working with older people who may not be in touch with professionals to encourage and develop conversations with them about suicidal thoughts that can help with support and signposting to further assessment. The results have significant implications for providing proper assistance for older adults and, as a result, minimizing suicide ideation. The research highlights the need to increase awareness of the variety of suicide manifestations at later ages and promote more sensitivity to how it may emerge. This understanding will increase the likelihood of identifying and reacting to expressions and behaviors associated with suicide.

The eleventh article by Zhao et al., entitled “*The relationship between gender, marital status, and depression among Chinese middle-aged and older people: Mediation by subjective well-being and moderation by degree of digitization,”* is an original research article that examined the association between depression, subjective wellbeing, degree of digitization, and socio-demographic variables, namely gender and marital status, among 15.586 middle-aged and older people from the 2018 national baseline survey data of the China Health and Retirement Longitudinal Survey (CHARLS). Findings of the mediating effect of subjective wellbeing between gender and depression, the mediating effect of subjective wellbeing between marital status and depression, and the moderating effect of degree of digitization between subjective wellbeing and depression highlighted the practical keys for policymakers and mental health therapists. The authors suggested improving middle-aged and older adults and promoting their integration.

The last article by Aisenberg and Harmatz, entitled “*Improving depressive symptoms and maintaining cognitive abilities of seniors within the nursing homes: A pilot study of brief mindfulness-based interventions for seniors in a semi-randomized trial,”* is an original research article that presents an intervention using a brief intervention on mindfulness for older adults in nursing homes. The study involves eight half-hour sessions, either with an 8-week course of weekly meetings or a 4-week course of two sessions per week, compared to a control care-as-usual group. Such brief interventions are important as current interventions are usually too long and costly, heavily dependent on counselors for delivery, and physically and cognitively demanding for older adults in nursing homes. The authors demonstrate that the brief interventions were promising in improving mindfulness, psychological distress, and, selectively, cognitive capacity. It potentially impacts providing a quick intervention treatment to older adults in enhancing their quality of life and wellbeing.

Regarding future study proposals, empirical investigations on older persons with various psychopathologies and other health problems associated with older adults, such as dementia, are recommended. Several studies on older adults have explored anxiety (Mowla et al., 2022) and depression (Richardson et al., 2020; Tyler et al., 2021; Mowla et al., 2022). However, those studies have examined socio-demographic aspects of psychopathologies in great detail. In future research, it is proposed to explore a variety of psychopathologies (other than depression and anxiety) with biopsychosocial variables. For instance, it can be observed that three of the papers contained in this collection concentrate on the problem of suicide among older people who reside on various continents. For the purpose of formulating preventative initiatives, it is generally agreed that research concentrating on the factors that influence suicide rates among older adults will be required.

This collection of research includes studies that investigate psychopathology and four studies that investigate the wellbeing of older adults. It is generally agreed upon that the issues related to the health and happiness of older adults are extremely significant for preventative measures. A few studies have highlighted how important it is for the wellbeing of older adults to have meaningful relationships with their peers. The value of intergenerational interactions as well as therapies that can be offered remotely to combat loneliness, have been emphasized throughout the volume. Implementing new strategies for promoting the wellbeing of older adults will necessitate using evidence-based trials in the future.

Karakose (2022) highlighted the importance of the quality of late-life marriages, which may directly associate with both spouses' mental health. Understanding the interpersonal and intrapersonal factors related to mental health issues within marriages may provide more resources to enhance the quality of their lives in older couples. Also, a recent study on spousal caregiving couples by Monin et al. (2019) mentioned that the health issues of both partners might uniquely impact caregivers' relationship satisfaction. Thus, future studies should investigate the aspects of relationship satisfaction related to one's own and the partner's health among older caregiving couples.

This Research Topic is limited to the topics in the articles listed above. We should mention that several factors have been linked to mental health issues in older adults. For example, personality factors, particularly neuroticism and conscientiousness, have been found to be closely associated with late-life depression and Alzheimer's disease and related dementias (Kotov et al., 2010; Hayward et al., 2013; Koorevaar et al., 2013; Terracciano et al., 2014, 2021; Sutin et al., 2018; Singh-Manoux et al., 2020; Aschwanden et al., 2021; Luchetti et al., 2021). From a broader perspective, psychological health strategies for older adults should also focus on identifying and addressing risk factors that are more prevalent in older adults, such as chronic physical conditions, inadequate social support networks, neglect and abuse, lack of transportation, financial barriers, or specific issues brought on by cultural differences, as well as how these risk factors contribute to the onset, maintenance, and long-term effects of mental health problems.

In conclusion, the prevalence of psychological disorders such as depression, anxiety, stress, psychosis, and substance abuse increases as the world's population ages. Thus, healthcare providers, researchers, and policymakers must recognize the importance of addressing mental health issues in older populations and work to develop effective, evidence-based strategies for preventing, assessing, and treating mental health conditions in this population that are specific to the needs of older adults.

## Author contributions

All authors listed have made a substantial, direct, and intellectual contribution to the work and approved it for publication.
